# Direct manufacturing of ultrathin graphite on three-dimensional nanoscale features

**DOI:** 10.1038/srep22700

**Published:** 2016-03-04

**Authors:** Mercè Pacios, Peiman Hosseini, Ye Fan, Zhengyu He, Oliver Krause, John Hutchison, Jamie H. Warner, Harish Bhaskaran

**Affiliations:** 1 Department of Materials, University of Oxford, Oxford, OX1 3PH United Kingdom; 2 Nanoworld Services GmbH, Schottkystraβe 10, 91058 Erlangen Germany

## Abstract

There have been many successful attempts to grow high-quality large-area graphene on flat substrates. Doing so at the nanoscale has thus far been plagued by significant scalability problems, particularly because of the need for delicate transfer processes onto predefined features, which are necessarily low-yield processes and which can introduce undesirable residues. Herein we describe a highly scalable, clean and effective, *in-situ* method that uses thin film deposition techniques to directly grow on a continuous basis ultrathin graphite (uG) on uneven nanoscale surfaces. We then demonstrate that this is possible on a model system of atomic force probe tips of various radii. Further, we characterize the growth characteristics of this technique as well as the film’s superior conduction and lower adhesion at these scales. This sets the stage for such a process to allow the use of highly functional graphite in high-aspect-ratio nanoscale components.

The manufacturing of large-area graphene has taken front-stage because of its potentially numerous applications (electronic, photonic, sensor, metrology, energy, materials, biological, etc.)^1^. Currently, there are probably a dozen methods being used and developed to prepare graphene of various dimensions, shapes and quality and several others that demonstrate its integration into functional devices^2^,^3^. The choice of the synthesis method depends on the end-application of the material^4^. For instance, the tip of an atomic force microscope probe is an ultrasharp nanoscale feature, and has been the focus of recent graphene-based work due to its interesting electrical and tribological properties^5^,^6^,^7^,^8^ viz, it has been shown to improve the performance of AFM probes compared to the existing commercial ones. Shim *et al.* created multifunctional cantilever-free multilayer graphene-coated scanning probe arrays that have wear-resistant tips of high sharpness and electrical and thermal conductivities^5^; Wen *et al.* used multilayer graphene sheets directly grown on Au coated tips as electrodes for robust molecular junctions with electronic behavior showing high performance in conduction mode^6^; Lanza *et al.* reported graphene single layer-coated commercial Pt-Ir tips that are extremely stable and resistant under high current and friction, leading to longer device lifetime^7^ and Gimzewski and co-workers demonstrated the fabrication of single layer graphene-SU-8 based AFM probes turning them conductive and more resistant to wear^8^. Chemical vapor deposition (CVD) of graphene was employed in all of the above as CVD of hydrocarbon gases has been demonstrated to be an attractive approach because of the ability to grow high-quality graphene films over a large area. These experiments underscored the potential impact of graphene in such applications, but the use of gaseous raw materials, high temperature processing or multi-step synthesis routes (transfer processes that lead to wrinkles, lack of cleanliness and scratches) make them impractical for large-scale manufacturing, particularly on pre-existing nanometric high aspect ratio structures; therefore, methods that enable *in-situ* synthesis on such structures are urgently required.

A scalable, clean and less hazardous technique is the catalytic graphitization of solid carbon sources. In this method, carbon is not precipitated from the gas phase as in CVD, but rather as a solid carbon film of finite thickness deposited below or atop a catalyst film. It is then possible to obtain mono-, few-layer graphene or ultrathin graphite at the catalyst surface or interface by thermal annealing, as has already been demonstrated on flat substrates^9^,^10^,^11^,^12^,^13^,^14^. Herein, we report on a simplified method using *in-situ* metal-catalyzed crystallization of amorphous carbon to ultrathin graphite on high aspect ratio nanometric features at lower temperatures such as 800 °C. This has allowed us to manufacture uniform and functional ultrathin graphite-probe tips (uG-AFM) in a one-step process as a model for a scalable approach. We further characterized the graphite to demonstrate that the epitaxial growth of ultrathin graphite on a nanoscale non-flat substrate is possible. Such *in-situ* growth would enable several applications (Fig. 1a) of graphene such as low friction and low-stiction micro- and nanoelectromechanical systems (MEMS and NEMS)^15^,^16^,^17^, micro/nanomachines^18^, bio-MEMS^19^, molecular electronics^20^ and even nanopatterning of graphene via controlled AFM tip exfoliation on a continuous basis^21^,^22^.

## Results

### Direct growth of ultrathin graphite (uG) on flat substrates without transfer

**Figure 1:**
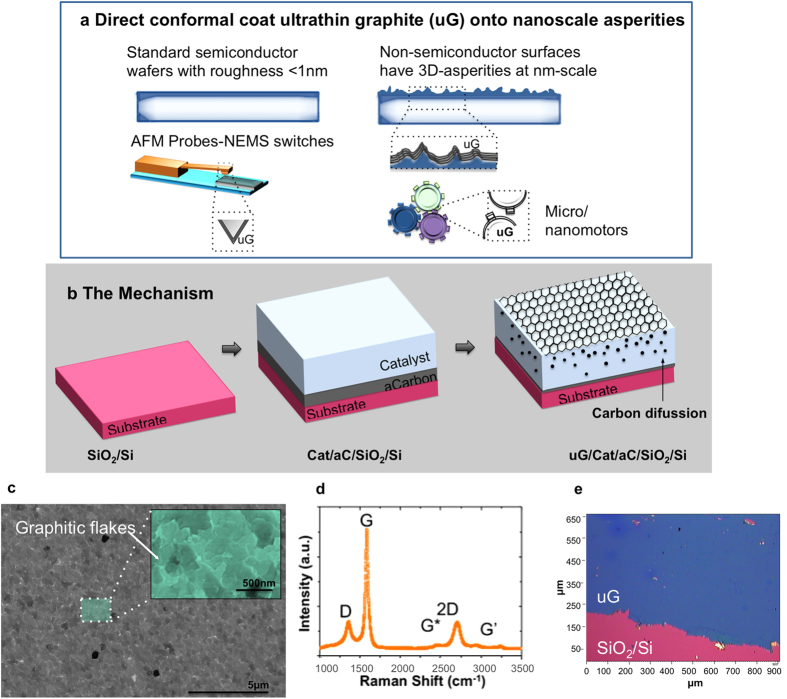
Direct-growth of ultrathin graphite (uG). (**a**) Potential applications on nanostructured components: Semiconductor processing uses ultra-flat wafers, but real surfaces for most other applications are not flat, and have many 3D features; therefore direct-growth will have a positive impact on AFM probes, MEMS, NEMS, molecular electronics on post-processed components with graphene/graphite to improve reliability. (**b**) Mechanism of growth on flat substrates without transfer. First, amorphous carbon and the metal catalyst are deposited on SiO_2_ by sputtering. Next, the film is annealed and ultrathin graphite is precipitated on the surface. (**c**) SEM micrograph showing homogeneous growth of graphene flakes on a flat SiO_2_ substrate to validate processes initially; (**d**) Raman characterization (at 532 nm excitation) of a ultrathin graphite film on the catalyst, Pt, showing characteristic graphitic peaks (on flat SiO_2_) (**d**) Optical microscope image of ultrathin graphite subsequently transferred on SiO_2_ substrate.

Figure 1b shows our schematic of the process for growing ultrathin graphite directly on a substrate without an intermediate transfer process. The fabrication process starts with the deposition of an amorphous carbon thin film beneath a catalyst film using sputtering. The sample is then inserted in a quartz tube furnace with argon flow and is annealed at 800 °C. (See the experimental section for more details). This causes the amorphous carbon to diffuse through the metal film due to high solubility of carbon in the metal. During the cool down, the carbon segregates and graphitic flakes are formed on the top surface as the solid solubility limit is reached. In Fig. 1c and e we show that the ultrathin graphite film has grown homogeneously thorough the entire substrate and that it is polycrystalline. We then carried out Raman spectroscopy to identify the graphene layers^23^,^24^.

As shown in Fig. 1d, there are three prominent peaks (D,G and 2D) and three small peaks (D + D″, D + D′, and 2D′). The D (≈1350 cm^−1^) peak is due to the breathing mode of six-atom rings and requires defects for its activation (“disorganized” carbon atoms in the samples or small graphite crystal size). G (≈1580 cm^−1^) derives from the graphene sheet (E_2g_ vibration mode active in all graphitic materials) and 2D (≈2710 cm^−1^) is the second order of D peak^24^,^25^. We observe that the intensity of G peak is larger than the one of 2D peak (I_(G)_ > I_(2D)_) as well as a broad and asymmetric 2D peak with small shoulder, all of which are characteristics of ultrathin graphite. Although our method is to enable direct growth, in order to initially carry out metrology on the ultrathin graphite, we transferred the uG onto SiO_2_/Si substrates. Transfer to SiO_2_ (300 nm)/Si substrates is carried out using an electrolysis-based bubbling method in NaOH (1M) aqueous solution to detach PMMA-supported graphite from the catalyst, with the PMMA subsequently removed in acetone^26^. Figure 1e shows an optical microscope image of the transferred ultrathin graphite film. There are a few imperfections, most likely caused during the transfer process. A thickness of ~20 nm was measured with an AFM.

### Ultrathin graphite growth on high aspect ratio features

**Figure 2:**
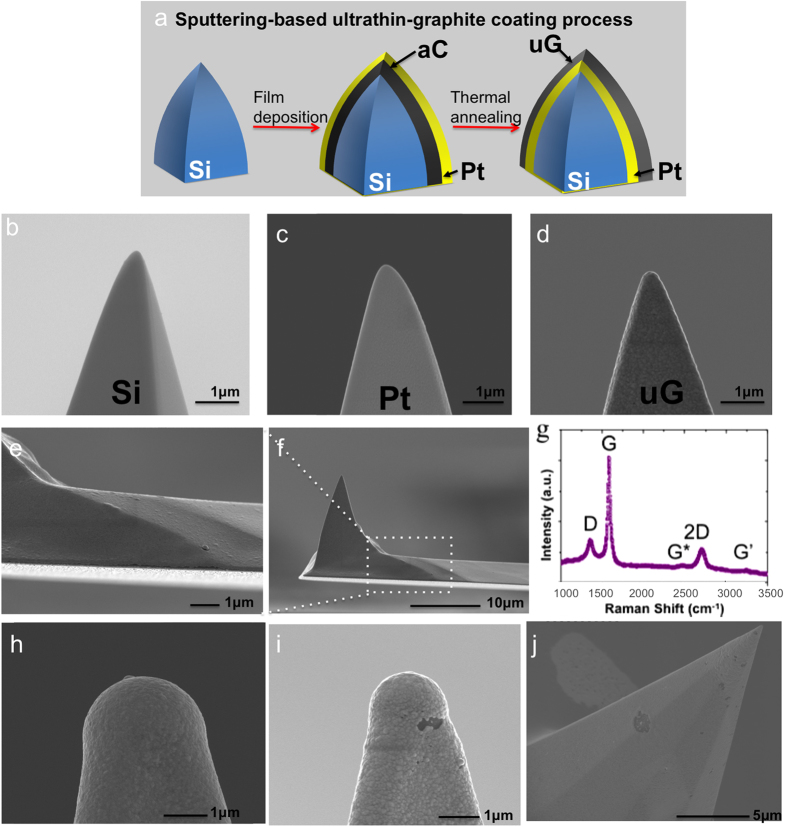
Ultrathin graphite growth on high aspect ratio features. (**a**) Schematic of growth on a 3-dimensional nanometric feature. SEM micrographs at each step of the fabrication of the ultrathin graphite coated AFM tips without transfer process: (**b**) AFM bare silicon tip, (**c**) coated with aC/Pt before annealing and (**d**) the tip coated with uG after annealing. (**e**,**f**) Detailed AFM probe after growth. Rounded tip, radius: 90 nm/150nm. (**g**) Raman Spectra (at 532 nm excitation) showing characteristic uG peaks at the tip. (**h**–**j**) SEM images of ultrathin graphite grown on different curved surfaces. Scaling down, diameter: (**h**) 4 μm, (**i**) 2 μm, (**j**) 30 nm respectively.

This growth technique can be highly scalable; graphitic layers are grown *in-situ* after sputter deposition of thin films, followed by annealing - all highly repeatable, robust and clean (since they take place in controlled atmospheres) processes. Importantly, such a thin film deposition procedure is compatible with structures with high aspect ratios (Fig. 2). To demonstrate this, we chose AFM tips of different diameters, from 4 μm down to a few nanometers, Fig. 2h–j (See Methods section for more details). AFM tips provide a perfect platform for testing such growth – they provide a 3-dimensional nanoscale feature - because they have been the focus of recent graphene-based work, mimic nanoscale electrical contacts in NEMS switches while also providing a very versatile platform for non-destructive TEM studies as we show later. There are several parameters that can affect the metal-catalyzed crystallization on those structures and thus the quality of the graphene layers, such as the catalyst, its structure, deposition method, annealing methodology, etc. (See Supplementary Table 1). We find that the best results are achieved using an amorphous carbon sputtered thin film (thickness 30 nm) beneath a sputtered platinum film as a catalyst (thickness 100 nm) annealed for 30 minutes at 800 °C under argon flow. Moreover, the coverage is less defective or less challenging with the larger diameter tips (>100 nm) (see Fig. 2) indicating that indeed there are changes to the growth process with very high aspect ratio structures where assuring proper coverage and a reduced stress of the catalyst film become key factors for the growth on such structures compared to the growth on flat surfaces.

It is well known that the catalyst plays a key role in the formation of graphene layers. In our particular system, platinum has given us better results in terms of surface morphology, sample homogeneity and graphite quality. Compared with the other commonly used catalyst metals for the epitaxial growth of graphene, Pt has a higher melting temperature and a lower thermal expansion coefficient that could reduce surface agglomeration and surface roughness during graphene synthesis at high temperature^27^. The sputtered film is mainly polycrystalline, single-oriented Pt (111), which minimizes the surface energy of the metal, as can be seen in the AFM image and XRD pattern of the Supplementary Fig. 1. The difference in the thermal expansion coefficient between Pt and graphene is smaller than other metals, which is potentially advantageous in reducing mechanical stress-induced defects in graphene on metal, especially if we aim to work with nanoscale curved surfaces. Moreover, unlike other metals such as Ni, which are more easily oxidized, Pt is inert, which can also reduce surface irregularities and maintains its catalytic capability.

In our case, sputter deposition of the amorphous carbon ‘seed’ layer and the catalyst directly on top was found to yield superior results when compared to thermal evaporation; in fact, we were not able to get clean, uniform and controllable growth when the carbon and the catalyst were evaporated. In thermal evaporation the carbon and the catalyst are deposited from different chambers, which would affect in the cleanliness of our system and therefore in the quality of the graphite grown. Moreover, evaporated materials deposit no-uniformly if the substrate has a rough surface. Sputtering both layers in the same deposition run had major advantages in the quality, in being able to have precise control of the properties of the film (by control of bias, pressure, etc.) and in that the deposition process, due to its poor directionality, has better step coverage, necessary for the 3D structures. These advantages of sputtering over thermal evaporation are significant for growth required on high aspect ratio nanometric structures such as nansocale tips and prefabricated NEMS.

**Figure 3:**
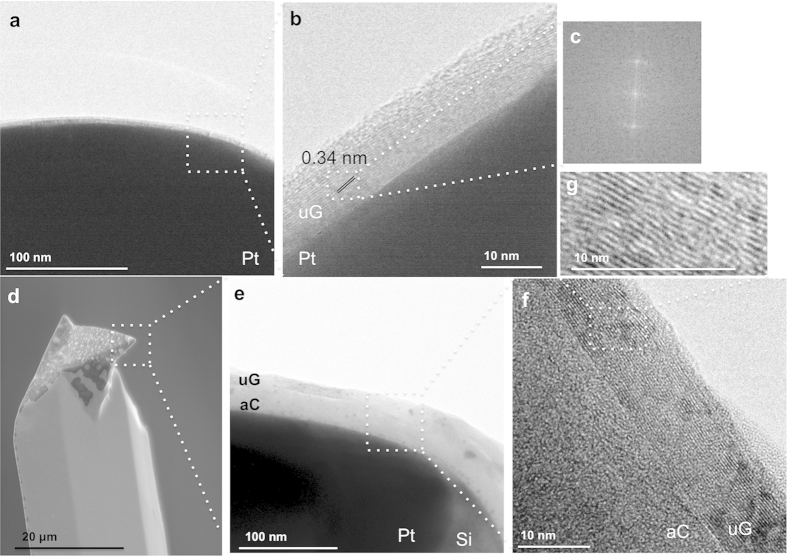
TEM characterization of the growth process. Detailed high-resolution TEM images of the edge of AFM tips after the growth process. The uG extends over the entire surface with thickness of ~10 nm. (**a**) uG grown by annealing under Ar flow. (**b**) Zoom of an area indicated in (**a**,**c**) Fourier of transform from the region marked in (**b**) showing spacing between graphite layers of 0.34 nm. (**d**–**g**) uG grown under high vacuum, ~10^−7^ Torr; (**d**) Agglomeration of the Pt is shown in SEM image, as well as an amorphous thick layer underneath the graphitic one in the TEM (**e**,**f**,**g**) images.

Using this technique, we found growth of ultrathin graphite on flat substrates to be successful and robust in many annealing conditions such as under argon flow (200 sccm, 9 Torr), under low vacuum (base pressure of 5.2 × 10^−3^ Torr) and under high vacuum (base pressure of 7.5 × 10^−7^ Torr) leading to higher degree of graphitization under lower working pressures. However, that was not the case with nanometric high-aspect ratio curved systems where the very low pressures caused surface stress on the curved regions and therefore agglomeration of the catalyst during the annealing (Supplementary Fig. 2). The result is uncontrolled growth where we found combinations of amorphous carbon and graphitic layers (as shown in Fig. 3e,f) or in some instances, only amorphous carbon.

In order to understand the nature of the growth on these nanometric surfaces, we performed scanning electron microscopy (SEM) of the AFM tip. From SEM we compare the coverage of the substrate before and after each step of the process (Fig. 2b–d) and we also evaluate if the surface is covered with some form of carbon after annealing (darker images), but we are unable to distinguish between amorphous and graphitic surfaces. As shown in detail in Fig. 2d, our probes are completely covered with a darker shade. To verify the nature of this material, we performed Raman spectroscopy (at 50× magnification, 1.18 μm spot size) and we confirmed that the surface is covered with ultrathin graphite. We obtained the characteristic peaks on the AFM probe on three different parts: chip, cantilever and tip (Fig. 2g). These peaks were the same as the ones we had obtained previously with the flat substrates (see Supplementary Fig. 3) and suggest similar mechanism for the formation of ultrathin graphite. We do note that our resolution is sufficient to prove that graphite is present on the cantilever, but the tips being extremely small, this would have some error.

We address this limitation in characterization using high-resolution transmission electron microscopy (TEM) at the tip. We found this to be an important and useful characterization technique for evaluating the quality of our graphene layers. Particularly in our case, this is a non-destructive procedure where no sample preparation is required for imaging. Instead we use a specialized TEM holder to obtain high-resolution TEM images of the tips (see experimental section for more details), which sheds further light on the nature of the growth of uG.

Upon annealing, the catalyst surface recrystallizes inducing grain growth^28^. Using TEM, we observe that large areas are covered with a homogeneous graphitic film ~10 nm thick and that the interlayer spacing is 0.34 nm, confirming the graphitic structure, as seen in Fig. 3a–c and we note that the process is repeatable for a set of annealing methodology, temperature and time, giving a growth rate around 0.006 nm/s. This is the case when the annealing is undertaken under argon flow, at a pressure of 9 Torr. When the annealing is carried out at very low pressures from ~10^−3^ to ~10^−7^ Torr we find that the growth has a different characteristic. This is shown in Fig. 3e,f, where, although we observe the same graphitic film, there is a thick layer of amorphous carbon directly beneath the graphitic layer, i.e. the carbon has diffused from beneath the Pt, without forming graphite throughout the surface. Catalyst agglomeration is also observed, differentiating the platinum from the silicon. This suggests the importance of the annealing pressure for such systems.

### Tip-surface interactions and conductivities of different AFM tip-systems

We then show that ultrathin graphite grown in this manner could have enabling applications in emerging areas of MEMS and nanomechanics such as NEMS switches, low-stiction surfaces on moving parts such as micromotors, where both stiction and contact reliability are major issues^29^,^30^,^31^. In order to do this, we compared tip-surface interactions and conductivities of different AFM tip-systems. We used the commercial silicon rounded tips of 90 nm/150 nm radius and force constant of 48 N/m, coated with different materials and treated in the same way (all annealed at same time and temperature) for better comparison.

**Figure 4:**
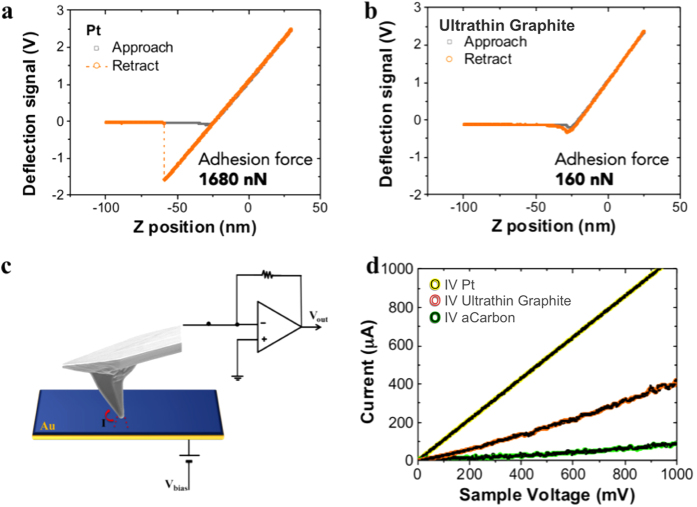
Tip-surface interactions and conductivities of different AFM tip-systems. Comparison of adhesion of a 90 nm/150 nm radius tips in air of (**a**) Pt coated APM tips and (**b**) ultrathin graphite tips on silicon substrate. Approaching (grey) and retract (orange) force curves. (**c**) Schematic and (**d**) I-V characteristics of tips with different coatings on Au using conducting AFM.

First, we systematically measured the interaction forces between a tip and a silicon substrate. In order to do that, we performed force-distance curves using atomic force microscopy. The main feature in Fig. 4a is the dramatic difference in the tip-surface interaction for a platinum coated tip compared to ultrathin graphite coated tip, Fig. 4b. As the cantilever is retracted from the surface, the tip remains in contact with the surface due to various surface forces, collectively influencing adhesion. We use the break-free distance as a measure of the adhesion force between the tips and the substrate. The distance was measured from the point that the tip feels a repulsive force to the point where the tip breaks away from the substrate (the jump in the force-distance curve). This force of adhesion can be estimated by multiplying the break-free distance by the spring constant of the cantilever. These experiments were carried out under ambient conditions, which is a well-known cause of high adhesive forces. The reason for this is that a thin layer of water and other condensed contaminants covers most samples in air. These contaminants often form a capillary bridge between the tip and sample, generating the large adhesive forces^32^. This serves as a good measure how much such a coating could reduce this adhesion. The adhesion force of the Pt- coated tip is ~1680 nN compared with the ~160 nN for the ultrathin graphite coated tip. Therefore, we observed that when the tip is coated with ultrathin graphite or amorphous carbon (not shown), the adhesion is reduced by almost a factor of 10 suggesting a hydrophobic character of the graphitic-based tips that reduce capillary forces in air. This is very encouraging as it suggests that this simple scalable process can be used to carry out post-processing on existing MEMS and NEMS components made using standard micromachining to increase their functionality and reliability.

Moreover, because no systematic variations were observed in the measured pull-off forces after repetitive trials, this also suggests that the MLG-AFM tips is a system were its durability would be of significant interest.

The second metric is the localized conductivity of our samples, useful for emerging systems such as NEMS switches and transistors^33^,^34^,^35^. To verify this, we performed contact resistance measurements on three different surfaces, viz. platinum, MLG and, amorphous carbon. Figure 4d shows the difference in the conductivity of Pt-coated, ultrathin graphite-coated and amorphous carbon-coated tips, decreasing in this order as a function of the voltage applied between the tip and the substrate. The high conductivity shown in the uG high aspect ratio system, together with the reduced adhesion forces makes it a potential system that could integrate electrical and mechanical functionality on the nanoscale features for graphene applications such as low-stiction MEMS and NEMS. This also confirms that the tip with graphite is essentially robust and maintains that coating layer in contact.

## Discussion

In conclusion, we have successfully demonstrated a process to directly grow ultrathin graphite on a continuous base on high-aspect ratio features at the nanoscale. The process consists of a one-step sputter deposition followed by annealing. This when applied to commercially available AFM tips of different diameters and sizes serves as a model for a clean, simple, inexpensive, less hazardous, reproducible technique that has the potential of being a large scale manufacturing methodology. We also have shown that the ultrathin graphite AFM tips have low adhesion forces in air and high conductivity. Therefore, this procedure could facilitate the direct integration of electrical and mechanical functionality on nanoscale features. For instance, MEMS/NEMS materials need to exhibit good mechanical and tribological properties on the micro/nanoscale as well as moving parts such as micromotors, where both stiction and contact reliability are major issues. In the field of AFM probes, low tip-sample interactions would be a benefit for those applications that require high stability or long-term operation. Low tip- sample forces are enabling in applications requiring reduction of the damage of the sample and probe and to avoid creating unwanted artifacts while imaging. Furthermore, because of the aromatic characteristic of graphene, such tips could be used as basis for biological engineering (i.e. Bio-MEMS). For instance, the ability to chemically modify small surfaces may open up new possibilities to study molecular electronic devices or biofunctionalized surfaces down to the nanoscale^19^.

## Methods

### Metal-catalyzed crystallization of amorphous carbon to ultrathin graphite

The AFM tips used were SD-Sphere-NCH and SD-R150-NCL form Nanosensors. Four different tips were evaluated (Spherical Silicon/Silicon dioxide tips of radius of 2 μm, 1 μm and 0.4 μm, force constant 42 N/m, 2.8 N/m and 0.2 N/m respectively; Rounded Silicon tips of radius of 90 nm/150 nm, force constant 48 N/m). The height of the tips was 10–15 μm. We used RF sputtering to deposit carbon and catalyst films directly onto a SiO_2_/Si flat substrate as well as on the AFM cantilevers at room temperature. The structure of the deposited films starting from the outermost layer was Pt (100nm)/C (30nm)/SiO_2_ (300nm)/Si. The sample was subsequently inserted in a quartz tube furnace equipped with an argon flow regulation system (or the same system equipped with rotatory pump for comparison). First the quartz tube was purged and filled with Ar gas for 30 minutes before the annealing process, which was also carried out in Ar atmosphere (9 Torr) we found this necessary for homogenous growth and to prevent potential oxidation of the sample. Then, the furnace was heated to 800 °C at a rate of 60 °C/min. Following this, the sample was inserted to the “hot-zone” and maintained at that temperature for 30 min. Finally, the sample was rapidly cooled to room temperature under Ar atmosphere by removing it from the “hot-zone” of the furnace. The typical Ar flow rate during the growth was 200 sccm.

### Characterization

The nanostructures of the films were analyzed by Raman spectroscopy: Micro-Raman (LabRam Aramis Horiba Jobin-Yvon), measurements were carried out with 532 nm excitation at 50× magnification, 1.18 μm spot size and 1.800 grating; X-ray diffraction (XRD, Philips MRD diffractometer); Transmission Electron Microscopy (TEM, Jeol 2100) was undertaken at an operating voltage of 200 KV and with a custom-made titanium holder which maintained the sample and the tip apex at specific Z height; Scanning Electron Microscopy (SEM, Jeol 6500F) was carried out at acceleration voltage of 5KV. Further characterization of the AFM tips (topography, current and force measurements) was carried out using the Asylum Research MFP-3D AFM (Oxford Instruments).

## Additional Information

**How to cite this article**: Pacios, M. *et al.* Direct manufacturing of ultrathin graphite on three-dimensional nanoscale features. *Sci. Rep.* **6**, 22700; doi: 10.1038/srep22700 (2016).

## Supplementary Material

Supplementary Information
