# Supplementary material for: Direct manufacturing of ultrathin graphite on three-dimensional nanoscale features
Source: Sci Rep. 2016 Mar 4;6:22700. doi: 10.1038/srep22700 (PMC4778042; doi:10.1038/srep22700)
Supplement: Supplementary Information [file srep22700-s1.pdf]

Supplementary Information

**Direct manufacturing of ultrathin graphite on three-dimensional nanoscale features**

*Mercè Pacios, Peiman Hosseini, Ye Fan, Zhengyu He, Oliver Krause, John Hutchison, Jamie H. Warner, Harish Bhaskaran**

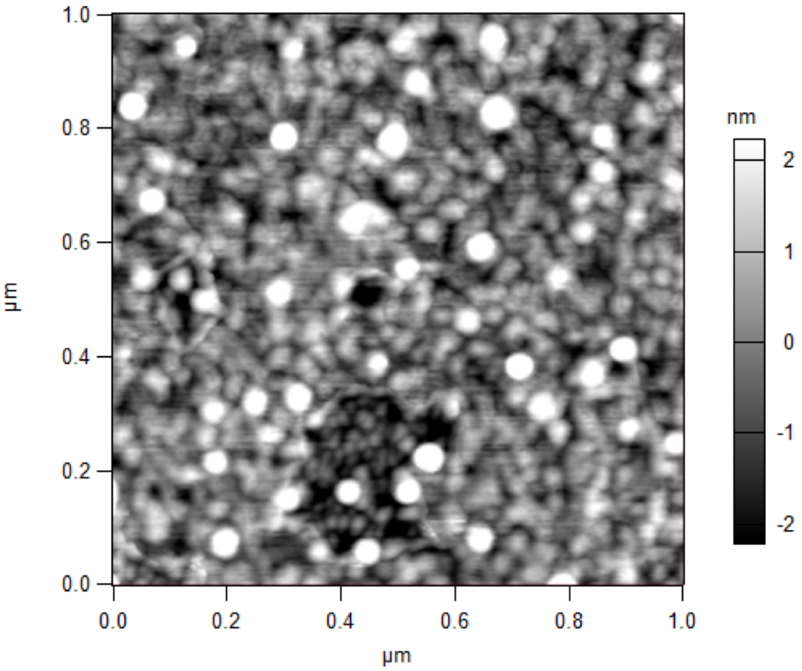


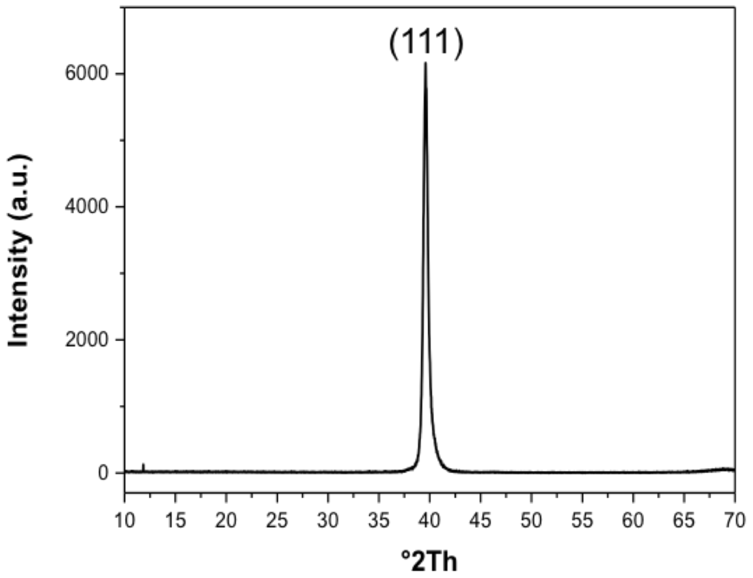


**Supplementary Figure 1:** AFM and XRD of the catalyst substrate. AFM topographical image and XRD pattern of 100 nm sputtered Pt film before annealing. AFM shows that the film is polycrystalline. The high and narrow single peak in the XRD pattern indicates that the film is Pt (111) oriented, which minimizes the surface energy of the fcc metal.

**Supplementary Table 1:** Optimization of growth parameters. Summary of some of the parameters explored for successful ultrathin graphite (uG) growth on flat substrates and high-aspect ratio nanometric substrates under particular processing conditions. Due to the growth methodology, we explored different catalysts with high carbon solubility (>0.1 atom %) such as cobalt[1], nickel[2, 3], platinum[4] (at high temperature >1135ºC) and palladium[5] varying the catalyst thickness (50-300nm), annealing methodology (different working pressures or under argon flow), annealing temperatures (750-900ºC), heating and cooling rates (5ºC/min denoted as slow rate and placing and removing the sample from the "hot zone" to ambient temperature before and after the growth, denoted as a fast rate), carbon thickness (15-50 nm) and deposition method of the catalyst and carbon (S, sputtering; E, evaporation). The best results were achieved using an amorphous carbon sputtered thin film (thickness ~ 30 nm) beneath a sputtered platinum film as a catalyst (thickness ~ 100 nm) annealed for 30 minutes at 800ºC under argon flow.

| Catalyst /substrate | Deposition method/ Thickness | Solid source | Deposition method/ Thickness | Annealing methodology | Temperature/ annealing time | Result (MLG?) |
|---|---|---|---|---|---|---|
| **Co/Flat** | E/100nm | Carbon | E/30nm | Furnace under Ar | 800ºC/30min Fast heating-cooling | No growth |
| **Ni/flat** | E/100nm | Carbon | E/30nm | Furnace under Ar | 800ºC/30min Fast heating-cooling | No growth Ni Oxidation |
| **Ni/Flat and AFM tip** | S/300nm | Carbon | S/30nm | Furnace under Ar | 800ºC/30min Fast heating-cooling | Amorphous carbon |
| **Pd/Flat** | S/100nm | Carbon | S/30nm | Furnace under Ar | 800ºC/30min Fast heating-cooling | No growth |
| **Pt/Flat** | S/50nm | Carbon | S/15nm | Furnace under Ar | 800ºC/30min Fast heating-cooling | No growth |
| **Pt/flat** | S/100nm | Carbon | S/50 nm | Furnace under Ar | 800ºC/30min Fast heating-cooling | Amorphous carbon |
| **Pt/flat** | E/100nm | Carbon | E/30 nm | Furnace under Ar | 800ºC/30min Fast heating-cooling | No growth |
| **Pt/Flat** | S/100nm | Carbon | S/30nm | Furnace under Ar | 750ºC/30min Fast heating-cooling | Low growth Weak Raman peaks |
| **Pt/Flat** | S/100nm | Carbon | S/30nm | Furnace under Ar | 800ºC/30min Fast heating-cooling | Homogeneous growth |
| **Pt/Flat** | S/100nm | Carbon | S/30nm | Furnace under Ar | 900ºC/30min Fast heating-cooling | Growth + Amorphous carbon |
| **Pt/AFM tip** | S/100nm | Carbon | S/30nm | Furnace under Ar | 800ºC/30min Fast heating-cooling | Growth on the tip |
| **Pt/Flat** | S/100nm | Carbon | S/30nm | Low vacuum $7\cdot10^{-3}$mbar | 800ºC/30min Fast heating-cooling | Homogeneous growth |
| **Pt/AFM tip** | S/100nm | Carbon | S/30nm | Low vacuum $7\cdot10^{-3}$mbar | 800ºC/30min Fast heating-cooling | No growth on the tip Catalyst agglomeration |
| **Pt/Flat** | S/100nm | Carbon | S/30nm | High vacuum $1\cdot10^{-6}$mbar | 800ºC/30min Slow heating/cooling (5ºC/min) | Homogeneous growth |
| **Pt/AFM tip** | S/100nm | Carbon | S/30nm | High vacuum $1\cdot10^{-6}$mbar | 800ºC/30min Slow heating/cooling (5ºC/min) | No growth on tip Catalyst agglomeration |

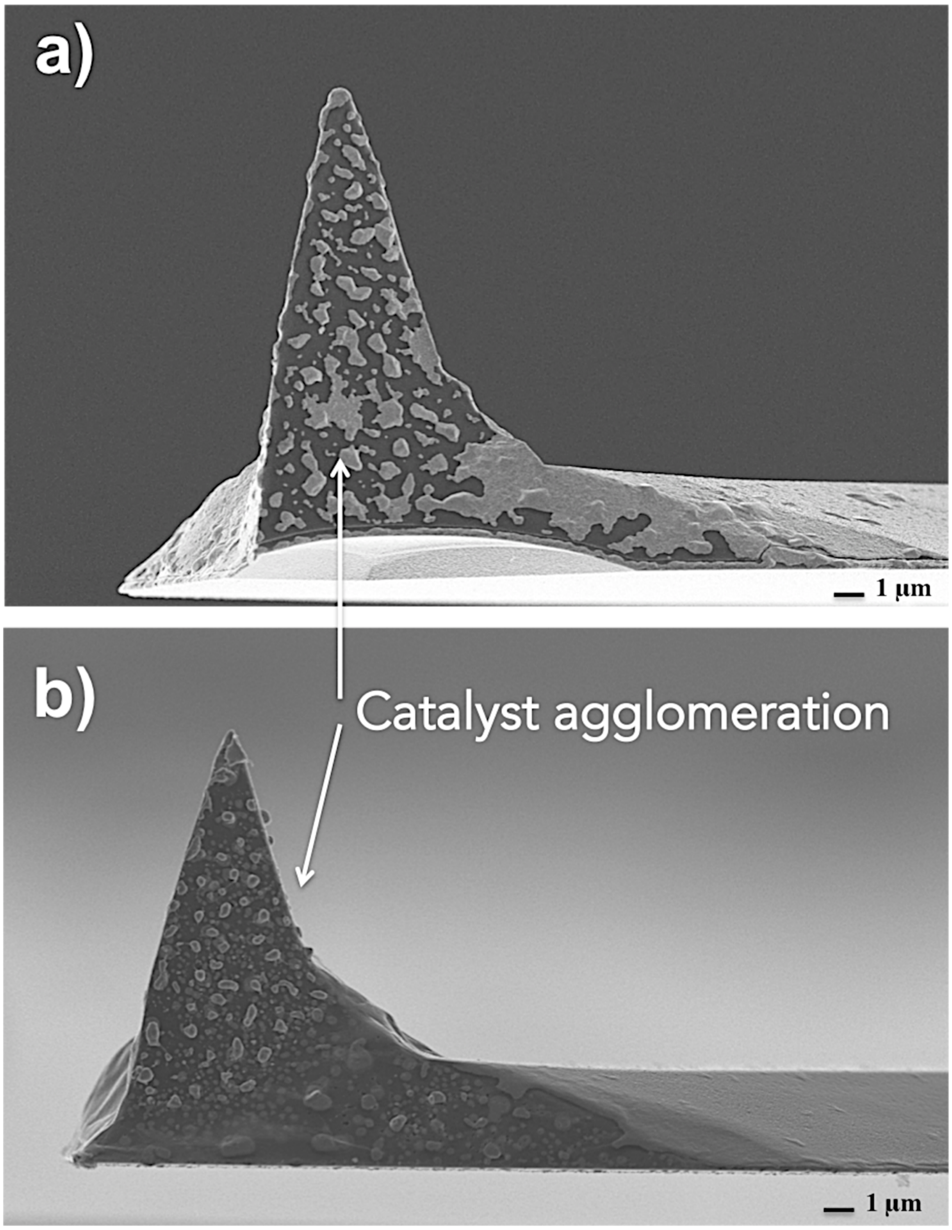


**Supplementary Figure 2:** Optimization problems: agglomeration on curved substrates. SEM pictures of AFM tips showing agglomeration of the catalyst at low pressures (From $1·10^{-3}$ to $1·10^{-7}$ Torr) on the high aspect ratio tips, but not on the rest of the cantilever. a) Sphere S tip, diameter of 0.8 µm, b) Rounded tip radius of 90nm/150nm

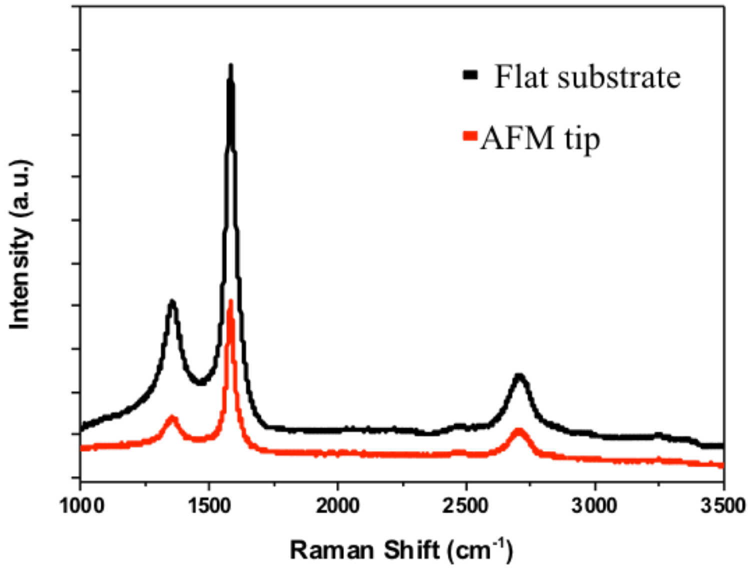


**Supplementary Figure 3:** Raman Spectroscopy on tip compared to that on a flat substrate. Comparison of Raman spectroscopy data of a flat substrate and an AFM cantilever grown at the same time under the same conditions. They show the same uG characteristic peaks. Excitation wavelength is at 532 nm.

## References


1. Weatherup, R. S. et al Introducing Carbon Diffusion Barriers for Uniform, High-Quality Graphene Growth from Solid Sources. *Nano Lett.* **13**, 4624-4631 (2013).
2. Lander, J. J., Kern, H. E. & Beach, A. L., Solubility and Diffusion Coefficient of Carbon in Nickel: Reaction Rates of Nickel ‑ Carbon Alloys with Barium Oxide. *J. Appl. Phys.* **23**, 1305-1309 (1952).
3. Isett, L. C. & Blakely, J. M., Segregation isosteres for carbon at the (100) surface of nickel. *Surf. Sci.* **58**, 397-414 (1976).
4. Martin, M. T. & Hudson, J. B., Surface diffusion of carbon on (111) platinum. *J. of Vac. Sci. Technol.* **15**, 474-477 (1978)
5. Siller, R. H., Oates, W. A. & McLellan, R. B., The solubility of carbon in palladium and platinum. *J. Less- Common Met.* **16**, 71-73. (1968).
